# Paraquat Degradation by Biological Manganese Oxide (BioMnO_*x*_) Catalyst Generated From Living Microalga *Pediastrum duplex* AARL G060

**DOI:** 10.3389/fmicb.2020.575361

**Published:** 2020-09-15

**Authors:** Jakkapong Thongpitak, Pamon Pumas, Chayakorn Pumas

**Affiliations:** ^1^PhD Degree Program in Environmental Science, Environmental Science Research Center, Faculty of Science, Chiang Mai University, Chiang Mai, Thailand; ^2^Department of Environmental Science, Faculty of Science and Technology, Chiang Mai Rajabhat University, Chiang Mai, Thailand; ^3^Department of Biology, Faculty of Science, Research Center in Bioresources for Agriculture, Industry and Medicine, Chiang Mai University, Chiang Mai, Thailand

**Keywords:** microalgae, photosynthesis, bio-oxidation, herbicide, catalyst

## Abstract

Paraquat is a non-selective fast-acting herbicide used to control weeds in agricultural crops. Many years of extensive use has caused environmental pollution and food toxicity. This agrochemical degrades slowly in nature, adsorbs onto clay lattices, and may require environmental remediation. Studies have shown that biosynthesized manganese oxide (BioMnO_x_) successfully degraded toxic synthetic compounds such as bis-phenol A and diclofenac, thus it has potential for paraquat degradation. In this experiment, *P. duplex* AARL G060 generated low (9.03 mg/L) and high (42.41 mg/L) concentrations of BioMnO_x_. The precipitated BioMnO_x_ was observed by scanning electron microscopy (SEM), and the elemental composition was identified as Mn and O by energy-dispersive x-ray spectroscopy (EDS). The potential for BioMnO_x_ to act as a catalyst in the degradation of paraquat was evaluated under three treatments: (1) a negative control (deionized water), (2) living alga with low BioMnO_x_ plus hydrogen peroxide, and (3) living alga with high BioMnO_x_ plus hydrogen peroxide. The results indicate that BioMnO_x_ served as a catalyst in the Fenton-like reaction that could degrade more than 50% of the paraquat within 72 h. A kinetic study indicated that paraquat degradation by Fenton-like reactions using BioMnO_x_ as a catalyst can be described by pseudo-first and pseudo-second order models. The pH level of the BioMnO_x_ catalyst was neutral at the end of the experiment. In conclusion, BioMnO_x_ is a viable and environmentally friendly catalyst to accelerate degradation of paraquat and other toxic chemicals.

## Graphical Abstract

BiOMnOx catalyst generated from living *Pediastrum duplex* AARL G060.

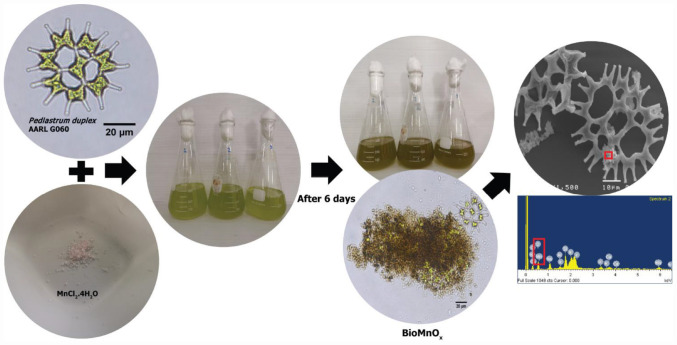

## Introduction

Non-selective herbicides, such as glyphosate and paraquat, were developed based upon their antagonistic effects on non-specific species of weeds and crops. Paraquat (1,1’-dimethyl-4,4’-bipyridinium dichloride) transforms electron flow from the photosystem and inhibits reduction of oxidized nicotinamide adenine dinucleotide phosphate (NADPC) during photosynthesis (Huang et al., 2019). Intensive paraquat usage over decades has led to widespread contamination of soil and water, and the World Health Organization (WHO) set an advisory limit for paraquat in drinking water at 10 μg/L.

Previous studies reported paraquat contamination in many countries worldwide (Huang et al., 2019). A paraquat concentration of 3.95 μg/L was reported in water samples from irrigation channels and rivers in Spain (Fernández et al., 1998). Paraquat residue in surface water in Brazil was found to be 0.279 μg/L (Veríssimo et al., 2018). Paraquat concentrations of 30.69–134.08 μg/L were detected in a stream in Mai Chau province, Vietnam (Thi Hue et al., 2018). Paraquat concentrations of 9.3–87.0 μg/L were reported in surface water in Thailand (Insuwan and Rangsriwatananon, 2017). In Malaysia, 0.6–6.9 μg/L of paraquat residue was detected in water samples (Ismail et al., 2011). These contaminants accumulate in the food chain creating public health hazards for humans, animals, and the environment (Frimpong et al., 2018).

To protect human health from the toxic effects of paraquat, removal is required. Biological methods may provide limited paraquat degradation (e.g., microorganisms utilize and degrade <1% of paraquat in soil particles (Roberts et al., 2002; Huang et al., 2019); therefore, abiological processes may be required to facilitate natural paraquat degradation. Previous studies included various methods for paraquat removal, such as adsorption on modified zeolites, activated carbon, and organoclay (Guégan et al., 2015; Sieliechi and Thue, 2015; Keawkumay et al., 2016; Pukcothanung et al., 2018). On the other hand, physicochemical processes using titanium dioxide, ozone, and various advanced oxidation processes showed potential for paraquat removal and reduction of the physical and chemical effects of this pesticide on the environment and health (Florêncio et al., 2004; Mandal et al., 2010; Wang and Xu, 2012; Deng and Zhao, 2015; Hamad et al., 2016).

The advanced oxidation process (AOP) is a conventional method for paraquat degradation based on the generation of highly reactive and non-selective hydroxyl radicals (∙OH) (Poyatos et al., 2009; Deng and Zhao, 2015; de Guimarães et al., 2016; Vagi and Petsas, 2017; Garrido-Cardenas et al., 2020). The high oxidative power of this radical can oxidize organic compounds to CO_2_ and H_2_O in aqueous solution. Fenton’s reaction is an AOP invented in 1894 when Fenton used ferrous ion and hydrogen peroxide as a reagent to improve tartaric acid oxidation (Nidheesh, 2015). Since then, this method has been widely used in wastewater treatment. The chemical process of this reaction is shown in Eqs. (1–3):

(1) $$H_{2}O_{2}+{\mathrm{Fe}}^{2+}\to {\mathrm{Fe}}^{3+}+{\mathrm{OH}}^{-}+∙\mathrm{OH},$$

(2) Organicmatter+∙OH→Oxidationintermediates,and

(3) $$\mathrm{Oxidation}\mathrm{intermediates}+∙\mathrm{OH}\to {\mathrm{CO}}_{2}+H_{2}O.$$

The reaction between iron and hydrogen peroxide produces hydroxyl radicals (eq. 1) with high oxidative potential. These radicals attack organic matter in wastewater (eq. 2) and generate oxidation intermediates which are further attacked by hydroxyl radicals to produce CO_2_ and H_2_O (eq. 3). However, Fenton’s reaction requires acidic conditions (i.e., a pH value in the range of 3–5 for high performance degradation of paraquat (Bishop et al., 1968; Kiwi et al., 1993; Barbusiñski and Filipek, 2001), and the acidic by-products are not environmentally acceptable. Thus, reactions to proceed at pH levels higher than that Fenton reaction.

Another non-acidic AOP is a “Fenton-like” reaction, a process that operates at nearly neutral pH. The pH value of the run-off effluent from this Fenton-like reaction is environmentally friendly and depends on other metals, such as copper, cobalt, manganese, and chromium, instead of iron (II), as catalysts for H_2_O_2_ decomposition (Nidheesh, 2015; Kim et al., 2017). The reaction of transition metals and hydrogen peroxide to generate hydroxyl radicals is shown in Eq. (4).

(4) Metal+HO2→2Metal+∙OH+OH.-

Various microorganisms can oxidize manganese, including bacteria, fungi, and algae (Greene and Madgwick, 1991; Li et al., 2019; Zhou and Fu, 2020). Previous studies revealed that some microalgae, such as *Desmodesmus* sp. WR1 and *P. duplex* AARL G060 increase dissolved oxygen content due to microalgal photosynthesis (Wang et al., 2017; Thongpitak et al., 2019). In addition, microalgae take up CO_2_ and HCO_3_^–^ from the culture medium on a cellular level. A decrease in H^+^ in the culture medium indirectly increased pH levels and generated microenvironments with pH values greater than 9 (Richardson et al., 1988; Richardson and Stolzenbach, 1995; Knauer et al., 1999; Chi et al., 2011). These mechanisms create conditions suitable for Mn oxidation as shown in Eq. (5):

(5) Mn+2+0.5Ofrom⁢microalgal⁢photosynthesis2+HO2→BioMnO.x

Use of microalgae to generate BioMnO_x_ has several advantages: it is environmentally friendly, and has higher efficiency and lower operation and maintenance costs than those of chemically (Li et al., 2019); however, some microalgae are sensitive to high manganese concentrations (Knauer et al., 1999).

Manganese oxide is a transition metal oxide used as a catalyst in various reactions (Corma et al., 2005; Asgari et al., 2020). It was found to be capable of oxidizing a wide range of recalcitrant compounds, including compounds with phenolic and fluoroquinolonic moieties and some antibacterial compounds (Wu et al., 2017). Manganese oxide has gained attention as a technologically important compound for degradation of toxins in the laboratory and in the field (Kim et al., 2012; Birkner et al., 2013; Robinson et al., 2013; Furgal et al., 2015; Kim et al., 2017). However, Mn oxides are prepared via chemical methods which require high energy and chemical reagents to convert the metal ions into precipitates (Fu et al., 2010; Shaker and Abdalsalm, 2018; Tian et al., 2018).

Recent research demonstrated the application of BioMnO_x_ produced by microorganisms for wastewater treatment (Sabirova et al., 2008; Hennebel et al., 2009; Forrez et al., 2010). Another paper reported that BioMnO_x_ can oxidize As(III) to As(V), which is a preliminary step for As removal (Tani et al., 2004). The removal of 17α-Ethinylestradiol (EE2) by oxidation in flow through bioreactors was achieved up to 57% using a BioMnO_x_ catalyst (Hennebel et al., 2009). In addition, BioMnO_x_ produced by green microalgae was used to degrade phenols and small organic molecules (Wang et al., 2017). For example, diclofenac, a non-steroidal anti-inflammatory drug, was removed by BioMnO_x_ at neutral pH. This was 10-fold faster than removal with chemically synthesized MnO_x_ (Forrez et al., 2010). However, the application of BioMnO_x_ as a catalyst in paraquat degradation was not investigated, so the objective of this experiment was to evaluate the feasibility of using BioMnO_x_ as a catalyst for paraquat (as model pollutant compound) degradation. The BioMnO_x_ was prepared using living microalga *P. duplex* AARL G060 via a biological reaction process. The results of this study demonstrate the feasibility of applying BioMnO_x_ for removal of harmful agents in the environment.

## Materials and Methods

### Living Microalga Culture of *Pediastrum duplex* AARL G060

Living green microalga *P. duplex* AARL G060 was obtained from the Applied Algal Research Laboratory (AARL), Department of Biology, Faculty of Science, Chiang Mai University, Thailand. Thongpitak et al. (2019) demonstrated that *P. duplex* can generate BioMnO_x_ at high concentrations of Mn, and so this algal strain was selected for use in this study. An axenic culture of microalgal stock was maintained in Jaworski’s Medium under the following condition: 72.51 μE⋅m^–2^⋅s^–1^ light intensity from a light emitting diode, 25°C ambient temperature, and continuous shaking at 130 rpm.

### Chemicals and Reagents

Chromatographic separation of paraquat was performed by application of high-performance liquid chromatography (HPLC). Paraquat (98% pure) was purchased from MilliporeSigma. Acetonitrile of HPLC grade was purchased from Merck KGaA. All other reagents were analytical grade.

### Paraquat Stock Solution

Paraquat stock solution, at a concentration of 1,000 mg/L, was prepared by dissolving 0.6910 g of paraquat in 1,000 mL of DW. The paraquat stock solution was then diluted to the desired concentration using simple dilution methods (Knepil, 1977).

### Natural Contaminated Wastewater

Water samples obtained from the surface of a rehabilitated lignite coal-mine reservoir in the northern part of Thailand (500292m E, 1966086m N). Water samples were collected in November and December 2017. Some physico-chemical parameters of the water sample were published elsewhere (Thongpitak et al., 2019). High-density polyethylene bottles of 20 L capacity were filled with natural wastewater and stored at 4°C prior to use; water samples were used to generate BioMnO_x_ using microalgae.

### Batch Experiment

#### Effect of Paraquat on Growth in *Pediastrum duplex* AARL G060

Living green microalga *P. duplex* AARL G060 were cultivated in water samples obtained from the rehabilitated reservoir. The microalga was cultivated with nutrients containing NaNO_3_ (0.09438 g/L), KH_2_PO_4_ (0.02606 g/L), CaHCO_3_ (0.0159 g/L), and MgSO_4_.7H_2_O (0.0500 g/L, Thongpitak et al., 2018). The initial concentration of paraquat was 10 mg/L. The initial microalgal optical density was set at 0.2 for each treatment. The batch culture of *P. duplex* AARL G060 was inoculated into 150 mL of modified medium in DW (LA-Pq, i.e., living microalga with paraquat) to compare to the control (modified medium in DW without paraquat). All treatments were performed in triplicate at ambient temperature and 111.81 mE m^–2^ s^–1^ light intensity using LED illumination. The microalga density was determined daily by measuring the OD_665_ using a Thermo Fisher Scientific^TM^ GENESYS^TM^ 20 Visible Spectrophotometer, and the pH value of the cultures was measured using a Starter 3100 pH Bench (Ohaus, United States). Living microalga cultures in flasks were shaken three times per day by hand to confirm that all algal cells were suspended and to prevent the dissolution of oxygen due to agitation which interferes with Mn oxidation during photosynthesis.

#### Production of BioMnO_x_ by *Pediastrum duplex* AARL G060

Manganese concentrations in this experiment were assigned the highest Mn concentrations found in the rehabilitated reservoir (20 mg/L, Thongpitak et al., 2019). The BioMnO_x_ nanoparticles were prepared via photosynthesis of *P. duplex* AARL G060 and designated as low or high concentration. At low BioMnO_x_ concentration (LA-Low-BioMnO_x_), the microalga was inoculated in 300 mL of natural wastewater containing a Mn concentration of 20 mg/L. For high BioMnO_x_ concentration (LA-High-BioMnO_x_), living microalga was inoculated in 900 mL of natural wastewater containing a Mn concentration of 60 mg/L, triple the concentration of low BioMnO_x_. After six days of cultivation, cell pellets containing BioMnO_x_ were collected by centrifugation at 3,500 rpm for 20 min. Next, samples were washed twice with a phosphate buffer at a pH of 8.00. Then washed with DW to remove residual Mn ions from the microalgal cells. The amount of precipitated BioMnO_x_ that remained on the cell pellets was dissolved with 10 mL of 1 mM EDTA at a pH of 3.40 and analyzed using the same method applied to Mn concentrations in the supernatant (Kenduzler and Turker, 2002; Webster et al., 2011; Thongpitak et al., 2019). The BioMnO_x_ concentration of the microalga was determined by atomic absorption spectroscopy (AAS).

#### Analysis of BioMnO_x_ Formation Using Scanning Electron Microscope With Energy Dispersive Spectroscopy (SEM-EDS)

The BioMnO_x_ catalyst produced by *P. duplex* AARL G060 was collected by centrifugation at 4,000 rpm. Cell pellets were fixed with 2.5% glutaraldehyde in 0.1 M of phosphate buffer at a pH of 8.00 overnight at 4°C. The samples were then washed with a phosphate buffer at a pH of 8.00. After that, the samples were dehydrated with an ethanol concentration series. Next step, the sample was mounted on stubs and thereafter gold-sputtered (Michalak et al., 2014). Characterization of BioMnO_x_ was observed using a JEOL-5410LV SEM equipped with an Oxford INCA EDS system to capture the distribution of the elemental composition of MnO_x_ displayed on the microalgal cell walls. The X-ray spectrum of each sample revealed the microelement composition.

### Paraquat Degradation by Fenton-Like Reactions Using BioMnO_x_ as a Catalyst

BioMnO_x_ generated by *P. duplex* AARL G060 for a duration of 6 days, was evaluated for its potential as a catalyst in paraquat degradation. The initial concentration of paraquat was 10 mg/L, which was mixed with the modified medium in DW in preparation for the following three treatments: 1) the control (i.e., the modified medium in DW), 2) the culture containing the living *P. duplex* AARL G060 with low BioMnO_x_ (LA-Low-BioMnO_x_), and 3) a culture containing living *P. duplex* AARL G060 with high BioMnO_x_ (LA-High-BioMnO_x_). Paraquat degradation was performed in 150 mL of culture in a 250 mL Erlenmeyer flask. Then, 13.5 mL of H_2_O_2_ was added to LA-Low-BioMnO_x_ and LA-High-BioMnO_x_ treatments following the method of Fang et al. (2017). During the experiment, samples from each treatment were collected at 0, 3, 6, 12, 24, 48, and 72 h by centrifuging at 3,500 rpm for 15 min to determine the amount of paraquat remaining. The pH was measured, and the paraquat degradation efficiency was calculated using eq. 6:

(6) $$\mathrm{Degradation}\mathrm{efficiency}\left(\%\right)=\left[1-\frac{C_{t}}{C_{0}}\right]\times 100,$$

where C_0_ is the initial paraquat concentration and C_t_ is the concentration at time t.

### Paraquat Determination by High Performance Liquid Chromatography

The HPLC was operated under the following conditions: the HPLC column was a VertiSep UPS-C18 analytical column (4.6 mm × 250 mm i.d., 5 μm) maintained at a temperature of 30°C. The mobile phase consisted of 0.14 mol sodium chloride and acetonitrile (60:40, v:v). All samples were eluted at a flow rate of 1 mL/min. The column eluent was monitored by HPLC at ambient temperature (25°C) using a Diode-Array Detection (DAD) detector (HP Series 1260, Agilent Technology). The wavelength was fixed at 257 nm. Calibration curves for the analysis were established with a standard solution of paraquat.

### Data Analysis

All of the graphs and statistical analyses were completed using Microsoft^TM^ Excel 2016.

## Results

### Effect of Paraquat on Growth of *P. duplex* AARL G060

**FIGURE 1:**
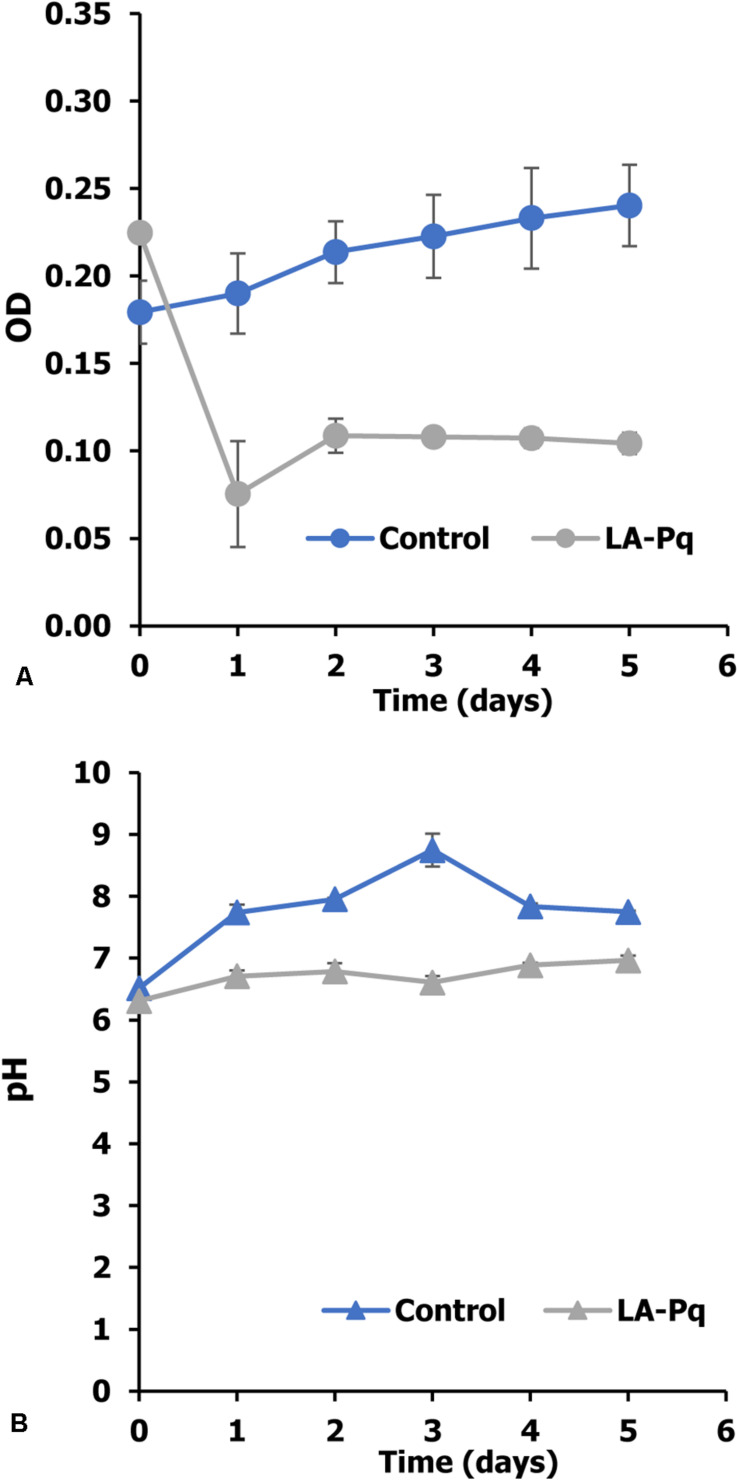
Effect of paraquat on growth of *P. duplex* AARL G060. **(A)** Optical density and **(B)** pH value. LA-Pq: living microalga with paraquat.

In this experiment, *P. duplex* AARL G060 was cultivated in a medium containing of N, P, C, and Mg to measure its growth (Thongpitak et al., 2019) using OD_665_. The results show that *P. duplex* AARL G060 grow well in the control medium (Figure 1A). The OD_665_ increased from 0.17 to 0.24. The growth of microalga also increased the pH level in the cultures; the highest pH level of a control treatment increased from 6.51 to 8.74 by the third day (Figure 1B). Meanwhile, the OD_665_ of the LA-Pq treatment decreased rapidly after the first day of cultivation (Figure 1A), and the pH level remained stable at approximately 6.80 until the end of the experiment.

### Biological Mn Oxide (BioMnO_x_) Generated by *P. duplex* AARL G060

**FIGURE 2:**
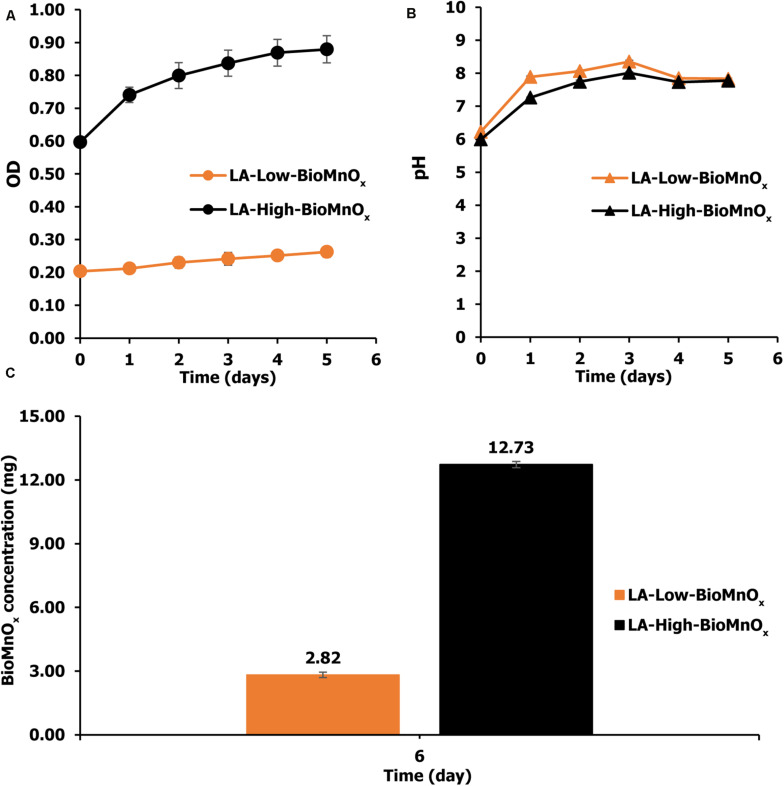
**(A)** Optical density, **(B)** pH value, and **(C)** concentration of BioMnO_x_ generated by *P. duplex* AARL G060. LA-Low-BioMnO_x_: living microalga with low BioMnO_x_ and LA-High-BioMnO_x_: living microalga with high BioMnO_x_.

The BioMnO_x_ catalyst was generated by *P. duplex* AARL G060 under the modified medium supplemented with Mn. Living microalgal growth was indicated by OD_665_). The oxide form of the Mn (MnO*_x_*) was achieved when the pH level in culture was raised. The results indicate that *P. duplex* AARL G060 grow well in both LA-Low-BioMnO_x_ and LA-High-BioMnO_x_ treatments (Figure 2A). The OD_665_ value increased from 0.20 to 0.26 on the last day, with the OD value of the LA-High-BioMnO_x_ treatment increased from 0.59 to 0.88 on the last day (Figure 2B), and the pH increased in both LA-Low-BioMnO_x_ and LA-High-BioMnO_x_ treatments to a level that supports Mn oxidation. The maximum pH level of LA-Low-BioMnO_x_ treatment was 8.35 on day 3. Similarly, the pH of the LA-High-BioMnO_x_ treatment was 8.01 on day 3. The BioMnO_x_ concentration in LA-Low-BioMnO_x_ treatment was 2.82 mg by day 6 (Figure 2C), and the BioMnO_x_ concentration for the LA-High-BioMnO_x_ treatment was 12.73 mg.

### Characterization of BioMnO_x_ Catalyst

**FIGURE 3:**
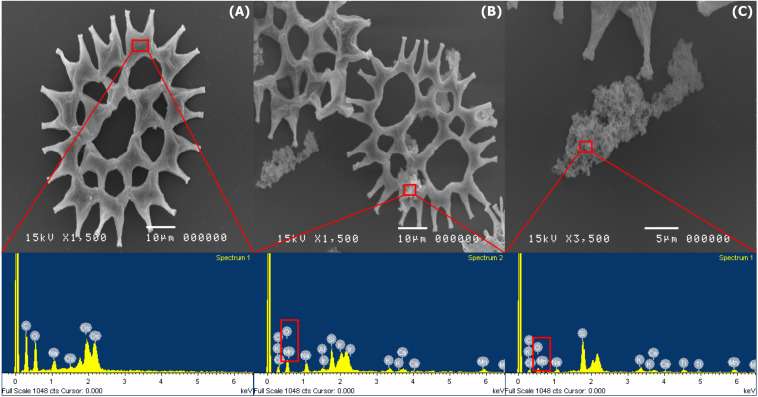
SEM microphotographs and EDS spectrums of BioMnO_x_ on the cell surface of *P. duplex* AARL G060. **(A)** Control and EDS spectra of selected area on cell surface. **(B,C)** Microalga cell and aggregate of BioMnO_x_ in culture and EDS spectra of selected area on day 6.

During the BioMnO_x_ process, the living microalgal culture presented dark brown particles of MnO_x_ on the cell surfaces which were investigated using the SEM. The microphotographs of *P. duplex* AARL G060 showed that Mn precipitation was not observed on the surface of the cells in the control group (Figure 3A). The cells of *P. duplex* AARL G060 were smooth and lacked Mn precipitation on cell surfaces. The SEM images of *P. duplex* AARL G060 with BioMnO_x_ are presented in Figure 3A. The results reveal that after 6 days of Mn generation, dark brown particles appeared in the microalgal culture. The brown deposits were only found as aggregates of algal cells with high levels of Mn. Aggregates of MnO_x_ were observed on cell surfaces in the SEM images shown in Figure 3B. Various sizes and irregular forms of BioMnO_x_ were presented on the microalgal cell surfaces. The relative elemental content of BioMnO_x_ was investigated using EDS. The EDS spectrum of the control treatment indicated the absence of Mn ions on microalgal cell surfaces. Mn was later detected in the EDS spectrum after 6 days of cultivation, and the BioMnO_x_ consisted of Mn and O in the complex (Figure 3C).

### Paraquat Degradation by Fenton-Like Reactions Using BioMnO_x_ as a Catalyst

**FIGURE 4:**
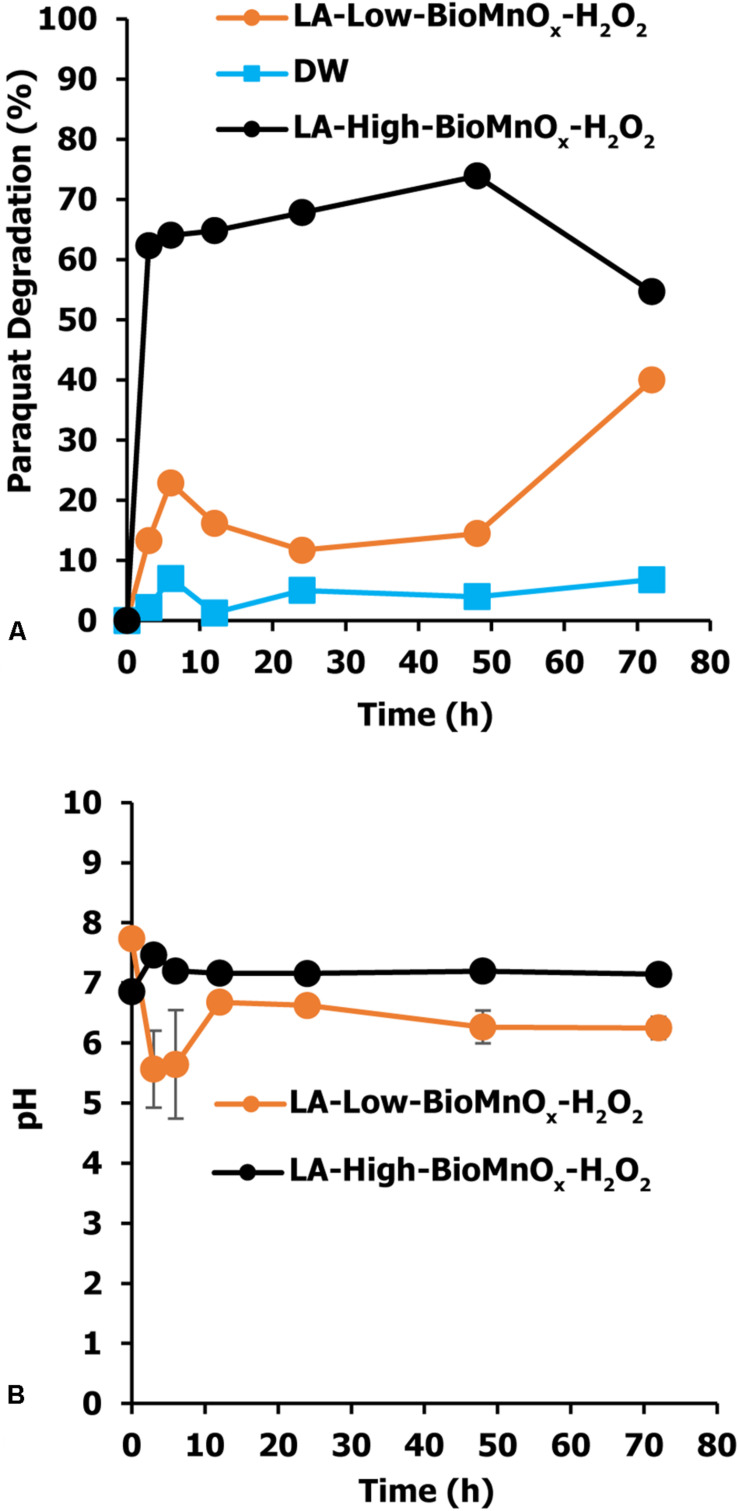
Paraquat degradation by Fenton-like reaction using BioMnO_x_ as catalyst. **(A)** Percent paraquat degradation and **(B)** pH level in paraquat degradation. LA-Low-BioMnO_x_-H_2_O_2_, living microalga with low BioMnO_x_ plus H_2_O_2_; DW, deionized water (control); LA, living microalga; CSMnO_x_-H_2_O_2_, chemical synthesis of MnOx plus H_2_O_2_; LA-High-BioMnO_x_, living microalga with high BioMnO_x_ plus H_2_O_2_.

**FIGURE 5:**
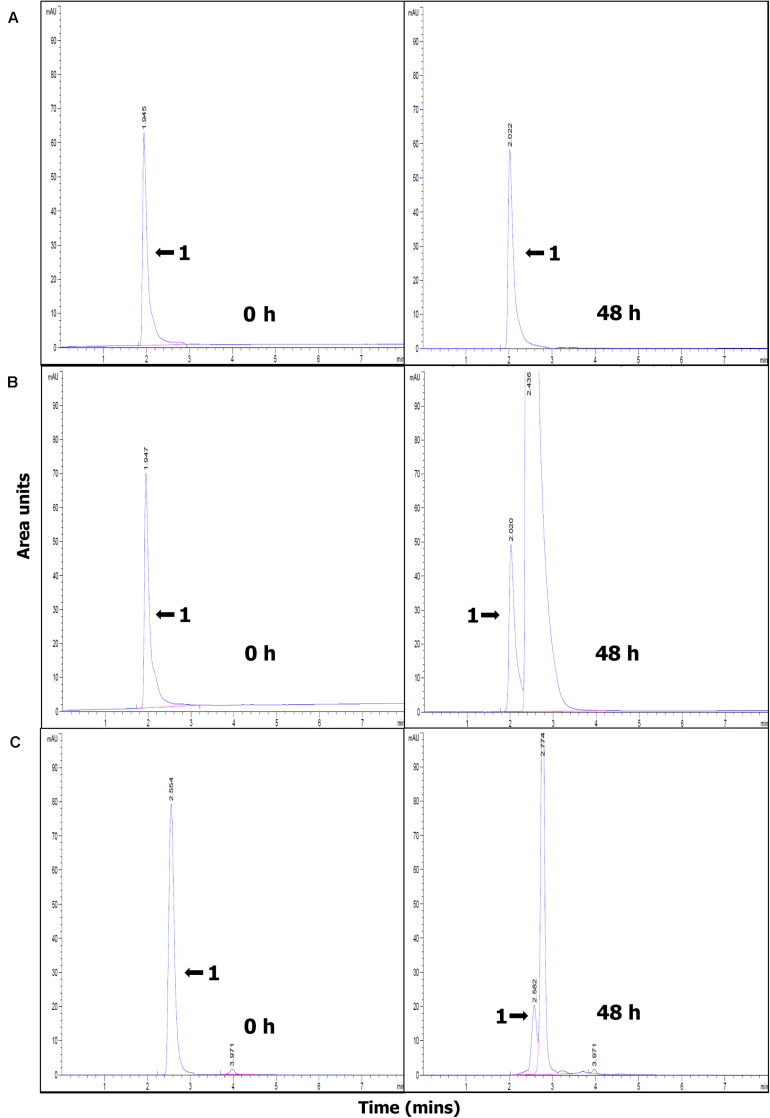
HPLC chromatogram showing paraquat degradation at 0 and 48 h. **(A)** DW (control), **(B)** LA-Low-BioMnO_x_-H_2_O_2_, and **(C)** LA-High-BioMnO_x_-H_2_O_2_ (the arrow with the number 1 indicates the peak representing paraquat). DW, deionized water; LA-Low-BioMnO_x_-H_2_O_2_, living microalga with low BioMnO_x_ plus H_2_O_2_; LA-High-BioMnO_x_, living microalga with high BioMnO_x_ plus H_2_O_2_.

The efficiency of paraquat degradation was studied starting with an initial paraquat concentration of 10 mg/L. The effect of BioMnO_x_ loading on paraquat degradation efficiency shows that the LA-High-BioMnO_x_-H_2_O_2_ treatment has higher paraquat removal efficiency than that of LA-Low-BioMnO_x_-H_2_O_2_ treatment (Figure 4A). This treatment degraded paraquat from 100 to 35.24% within 12 h of contact time and degraded up to 54.64% within 72 h. The results show that catalyst loading had a considerable effect on paraquat degradation efficiency, and that paraquat degrading efficiency improved as the loading increased. The LA-Low-BioMnO_x_-H_2_O_2_ treatment degraded paraquat from 100 to 83.84% within 12 h of contact time. Meanwhile, the control (DW) treatment maintained fairly stable paraquat concentrations. The pH level was above 5 from the initial time to the last hour of contact time (Figure 4B), and the HPLC chromatogram shows that the paraquat was degraded (Figure 5).

### Kinetic Study of Paraquat Degradation

A kinetic study of paraquat degradation in a Fenton-like reaction using BioMnOx as a catalyst was performed, and the pseudo-first order and pseudo-second-order kinetic models were applied to model the kinetics of paraquat degradation under different BioMnO_x_ concentrations. Each reaction proceeded at different rates. The pseudo first-order model is represented by Eq. (7):

(7) $$C_{t}=C_{0}exp\left(-k1.t\right),$$

where C_0_ and C_*t*_ represents are initial paraquat concentration and concentration at time t, k_1_ is the rate of degradation (constant) and the time t. Events for the pseudo-second-order model were described by Eq. (8):

(8) $$C_{t}=\frac{{\text{C}}_{0}}{1+{\text{k}}_{2}.\text{t}.{\text{C}}_{0}},$$

in which, k_2_ is the constant rate of degradation.

The results of the kinetic study indicate that paraquat degradation in LA-Low-BioMnO_x_-H_2_O_2_ treatment was fast during the initial period of 0–6 h, and the highest R^2^ value for this treatment was described as pseudo-first order and pseudo-second order kinetic model (Table 1). This first phase showed that the kinetic rate constants k_1_ and k_2_ were 0.0441 h^–1^ and 0.006 L.mg.h^–1^, respectively. The degradation rates in first period were faster than paraquat degradation in the Fenton-like reactions. For the LA-High-BioMnO_x_-H_2_O_2_ treatment, paraquat degradation was highest during the first period (0–6 h). This treatment could be described as pseudo-first order model because the k_1_ value was 0.2012 h^–1^. Higher constant rates revealed in this treatment demonstrated paraquat degradation efficiency higher than that of the LA-Low-BioMnO_x_-H_2_O_2_ treatment. Meanwhile, in a later period (6–72 h), the reaction of both treatments showed low degradation rates, which indicates a slower reaction.

**TABLE 1:** Kinetic parameters for Fenton-like oxidation of paraquat.

	Time (h)	Pseudo-first order k_1_ (h^–^^1^)	*R*^2^	Pseudo-second order k_2_ (L.mg.h^–^^1^)	*R*^2^
LA-Low-BioMnO_x_-H_2_O_2_	0–6	0.0441	0.9986	0.0060	0.9997
	6–72	0.0062	0.7876	0.0007	0.4560
LA-High-BioMnO_x_-H_2_O_2_	0–6	0.2012	0.9137	0.0284	0.8035
	6–72	0.0203	0.5803	0.0003	0.0254

## Discussion

The results from this study indicate that paraquat affects the growth of *P. duplex* AARL G060. The growth of microalga in LA-Pq treatment decreased with time. A previous study reported that paraquat accepted electrons from photosystem I, thus preventing electron transport to NADPH, and this action may block photosynthesis (Sétif, 2015; Reczek et al., 2017; Huang et al., 2019). Free radicals also react with oxygen, yielding superoxide anions, which led to the formation of hydrogen peroxide and hydroxyl radicals. The hydroxyl radicals caused changes in the ultrastructure of the cells and damaged the DNA in the microalgae (Qian et al., 2009; Zhang et al., 2014).

Living *P. duplex* AARL G060 converted Mn ions into solid Mn via photosynthesis and the oxidation process. After 6 days of cultivation, BioMnO_x_ was recorded on cell walls of the microalga. Photosynthesis of the living microalga increased the pH level of the water and produced oxygen. The oxygen was released to the environment to oxidize Mn ions and turn them into MnO_x_ on the microalgal cell surfaces, appearing as a dark brown solid aggregates (Taguchi, 1976; Richardson et al., 1988; Renger and Wydrzynski, 1991; Richardson and Stolzenbach, 1995; Bohutskyi et al., 2016). The results are consistent with previous studies which found that *Scenedesmus subspicatus* oxidized Mn, and MnO was observed both intracellularly and extracellularly (Knauer et al., 1999). In addition, the green microalga *Desmodesmus* sp. WR1 generated 13 mg/L of BioMnO_x_ after 3 days from an initial Mn concentration of 30 mg/L (Wang et al., 2017). The different quantities of Mn oxide depend on the level of oxygen production, the pH value, the number and size of microalgal cells, and the growth rate of each microalga species. For bacteria, previous studies revealed that the Mn-oxidizing bacterium *Aeromonas hydrophila* strain DS02 had a high tolerance for Mn(II) stress and generated up to 240 mg/L of Mn oxide in 6 days. The BioMnO_x_ coupled with peroxymonosulfate (PMS) activation degraded 99.5% of 2,4-dimethylaniline within 80 min (Zhang et al., 2019). Stuetz et al. (1996) reported that algal-bacterial oxidation by *Haemaetococcus* sp., *Chlamydomonas* sp., and *Chorella* sp. generated 210 ± 0.04, 170 ± 0.05, and 180 ± 0.05 mg/L of BiOMnO_x_ in 30 days, respectively (Table 2).

**TABLE 2:** BioMnO_x_ productivity of *Pediastrum duplex* AARL G060 compared with previous studies.

Microorganisms	Initial Mn concentration	BioMnO_x_ (mg/L)	No. of days	BioMnO_x_ productivity (mg/L/day)	References
*Desmodesmus* sp. WR1	30 mg/L (Mn^2+^ stock solution)	13.00	3	4.32	Wang et al., 2017
*Aeromonas hydrophila* strain DS02	1,258 mg/L (10 mM) (MnCl_2_)	240.00	6	40.00	Zhang et al., 2019
Algal-bacterial oxidation					Stuetz et al., 1996
*Haemaetococcus* sp.	5 g/L (MnSO_4_.H_2_O)	210.00 ± 0.04	30	7.00	
*Chlamydomonas* sp.	5 g/L (MnSO_4_.H_2_O)	170.00 ± 0.05	30	5.00	
*Chorella* sp.	5 g/L (MnSO_4_.H_2_O)	180.00 ± 0.05	30	6.00	
*P. duplex* AARL G060	20 mg/L (MnCl_2_.4H_2_O)	9.03	6	1.50	This study
*P. duplex* AARL G060	60 mg/L (MnCl_2_.4H_2_O)	42.41	6	7.06	This study

The application of chemical paraquat degradation methods discovered that the chemical process is a Fenton-like reaction that relies on metals, such as cerium, chromium, manganese, cobalt, and copper, to directly decompose H_2_O_2_ into ∙OH (Bokare and Choi, 2014; Nidheesh, 2015). A biological process characteristic of a Fenton-like reaction was also involved in the generation of the biocatalyst BioMnO_x_ by living microalga. The MnO_2_ generated showed potential as a strong oxidant that can transform aqueous pollutants according to (eq. 9):

(9) $${\mathrm{MnO}}_{2}+\mathrm{org}/\mathrm{inorganic}\,\mathrm{substrate}\to {\mathrm{Mn}}^{2+}+\mathrm{oxidized}\,\mathrm{organic}/\mathrm{inorganic}\,\mathrm{substrate}.$$

Then, a Fenton-like reaction, involving Mn ions proceeded as shown in Eqs. (10–13):

(10) $${\mathrm{Mn}}^{3+}+H_{2}O_{2}\to {\mathrm{Mn}}^{4+}+∙\mathrm{OH}+{\mathrm{OH}}^{-},$$

(11) $${\mathrm{Mn}}^{4+}+H_{2}\,O_{2}\to {\mathrm{Mn}}^{3+}+{\mathrm{HO}}_{2}^{∙}+H^{+},$$

(12) $${\mathrm{HO}}_{2}^{∙}\leftarrow \to H^{+}+O_{2}^{∙-},\mathrm{and}$$

(13) $$\mathrm{Pq}+∙\mathrm{OH}+O_{2}^{∙-}\to \mathrm{degradationproducts}.$$

Interconversion between Mn^2+^ and Mn^4+^ through intermediate Mn^3+^ species should allow the Mn-catalyzed Fenton-like activation of H_2_O_2_. The advantages of MnO_x_ oxidation have received intensive attention due to the large application area and environmental friendliness (Zhang et al., 2014).

The biological catalyst in the LA-Low-BioMnO_x_ treatment easily degraded paraquat in first period (0–6 h). The kinetics of the Fenton-like reaction during this initial period was described as pseudo-first and pseudo-second order kinetic models. During this period, the Fenton-like reaction used Mn ions which could increase reaction rates. During the last period (6–72 h), this treatment showed slower reaction rates and a decrease in paraquat degradation. The reason for this phenomenon is unclear but may be explained as follows. First, when *P. duplex* AARL G060 synthesized BioMnO_x_, Mn particles were generated both intracellularly and extracellularly (Thongpitak et al., 2019). Next, in two degradation steps, Mn^3+^ and Mn^4+^ from Mn oxide on cell surfaces reacted with H_2_O_2_ and generated OH which degrades paraquat. After that, when the microalga died, intercellular metal ions were released into solution. The Mn ions most abundant in solution were water-soluble Mn^2+^ and Mn^3+^ compounds. In aerobic neutral pH conditions, the oxidation of Mn^2+^ to Mn^4+^ and interconversion between Mn^2+^ and Mn^4+^ via intermediate Mn^3+^ species enabled the Mn-catalyzed Fenton-like activation of H_2_O_2_ (Bokare and Choi, 2014).

The results of the LA-High-BioMnO_x_ treatment also demonstrated the ability to degrade paraquat in solution by degrading more than 50% of the paraquat within 72 h. In addition, the amount of BioMnO_x_ catalyst produced had a positive effect on process efficiency. The initial period (0–6 h) of this treatment showed a high kinetic rate constant (0.2012 h^–1^). This degradation percentage of paraquat in LA-High-BioMnO_x_ was higher than that of LA-Low-BioMnO_x_ treatment during this period. During the last period (6–72 h), the kinetic rate constant was lower than the first period and also indicated that degradation rates stabilized in this phase. Even for the amount of BioMnO_x_ catalyst loading observed, the results are consistent with previous studies that attributed catalyst loading to an increase in the active substances; which, in turn, form more active radicals to contact the target pollutant (Fang et al., 2017; Abdellah et al., 2018; Sabouni and Gomaa, 2019), consistent with previous studies. Application of manganese oxide removed approximately 78% of bisphenol A within 168 h (Wang et al., 2017). Moreover, manganese oxide was used as act a catalyst in a Fenton-like reaction to completely degrade methylene blue in a short duration of 20 min (Kim et al., 2017). In addition, the co-synthesized Fe_3_O_4_-MnO_2_ nano-complex removed up to 96.8% of acid orange 7. The MnO catalyst performed better and degraded more toxin than either Fe_3_O_4_ or MnO_2_ alone.

Paraquat degradation efficiency depended on the dispersions of the catalyst. The highly aggregated BiOMnO_x_ on microalgal cell surfaces are shown in Figure 3. The nanoparticle catalyst may be well-dispersed that could support paraquat degradation. The size of BioMnO_x_ nanoparticles varied according to different Mn ion concentrations, microalgae concentrations, and cultivation time. Other than using viable microalgal cells, cell free extracts were also possible to biosynthesize for nano MnO (Kumar et al., 2017). In Kumar’s study, the size of nanoparticles was varied by alteration of metal ion concentrations, cell free extract concentrations, metal ion volume, cell free extract volume, and incubation temperature.

The observed paraquat degradation by the Fenton-like LA-High-BiOMnO_x_-H_2_O_2_ treatment after 72 h decreased. Regeneration of the catalyst could help degradation efficiency remain nearly constant. Cao et al. (2009) revealed a highly effective method for recovering the iron catalyst from Fenton and Fenton-like reactions in which samples were dewatered, dried, and baked at 350–400°C for 20–30 min. For the Mn catalyst, Poyraz et al. (2016) studied the recycling of manganese oxide cathodes for lithium-based batteries and found that thermal regeneration was a suitable method for recycling catalysts.

The HPLC profiles showed rapid disappearance of the peak corresponding to paraquat, accompanied by the appearance of new peaks. Although degradation products of paraquat were not evaluated in this study, degradation products from AOPs were suggested elsewhere. The degradation products may correspond to demethylation products such as 4,40-bipyridine and monopyridone and hydroxylation or oxidative ring cleavage products such as 4-carboxy-1-methyl-pyridinium ion, 4-picolinic acid, and hydroxyl-4-picolinic acid, in accordance with paraquat degradation by UV-ozonation (Kearney et al., 1988), or degradation products such as 4-carboxy-1-methylpyridinium ion, paraquat pyridine, and paraquat dipyridone as suggested by Florêncio et al. (2004) who studied paraquat degradation by titanium dioxide photodegradation. And Burrows et al. (2002) showed that irradiation of paraquat in the presence of oxygen led to formation of 4,4-bipyridyl and 4-picolinic acid. The degradation products of paraquat via Mn catalyzed Fenton-like reaction were not investigated in this study, but this issue should be considered in future studies.

The Fenton-like reactions using BiOMnO_x_ as a catalyst to remove contaminants from real-life full-scale wastewater provides a viable environmentally friendly alternative technology for the treatment of industrial wastewater.

## Conclusion

Based on the results of this study, *P. duplex* AARL G060 was found to be a potent strain of living green microalgae for BioMnO_x_ production. The BioMnO_x_ served as a biocatalyst that degraded 54.64% of the paraquat in aqueous solution, and the pH level of this operation was above 5. This result demonstrates that BioMnO_x_ is an environmentally friendly alternative catalyst to remove toxins in wastewater. The insights gained from this experiment will be used to develop better treatments for degradation of toxins and remediation of wastewater.

## Data Availability Statement

All datasets generated for this study are included in the article/Supplementary Material.

## Author Contributions

All authors listed have made a substantial, direct and intellectual contribution to the work, and approved it for publication.

## Conflict of Interest

The authors declare that the research was conducted in the absence of any commercial or financial relationships that could be construed as a potential conflict of interest.
